# Acute Kidney Injury Secondary to Vitamin D Intoxication: A Case of Oxalate Nephropathy

**DOI:** 10.7759/cureus.96305

**Published:** 2025-11-07

**Authors:** Millie Prime, Innocent Segamwenge, Aml Mousa

**Affiliations:** 1 Nephrology, Liverpool University Hospitals, Liverpool, GBR; 2 Pathology, Liverpool University Hospitals, Liverpool, GBR

**Keywords:** acute kidney inury, crystalline nephropathy, hypercalcaemia, hypercalciuria, nephrocalcinosis, oxalate crystals, renal injury, renal pathology, vitamin d toxicity

## Abstract

In recent years, vitamin D supplements have gained popularity in the media, leading more people to start taking them on their own. Vitamin D excess and subsequent intoxication are an uncommon but recognised cause of acute kidney injury. Excess of vitamin D leads to hypercalcaemia with a suppressed parathyroid hormone. Hypercalcaemia leads to hypercalciuria, which in turn causes supersaturation of urine with calcium salts. Deposition of these salts can create a form of acute kidney injury characterised by tubular obstruction and interstitial injury, as seen on a renal biopsy.

We report the case of a male in his 70s who presented with confusion and weakness. He was found to have a significant acute kidney injury with a metabolic acidosis. His serum-adjusted calcium was elevated, and he had an elevated phosphate and potassium. His parathyroid hormone level was suppressed, and a myeloma screen was negative. He was initiated on haemodialysis. It was later revealed by a family member that he had been taking large doses of vitamin D daily for more than three years, believing it would boost his immunity during the COVID-19 pandemic. Renal biopsy demonstrated diffuse acute tubular injury with clear refractile crystals within the tubules. The interstitium showed oedema and acute inflammatory infiltrate. He was commenced on prednisolone to suppress further activation of vitamin D and to treat interstitial nephritis. This led to an improvement in his renal function, and dialysis was discontinued. His renal function stabilised, but only a partial recovery was observed.

Excessive intake of vitamin D is associated with toxicity and long-lasting consequences. This may be patient-directed or secondary to drug prescription errors. Hypercalcaemia with suppressed parathyroid hormone and hypercalciuria are hallmarks of such toxicity, as seen in our patient. The histopathology in our case showed radially arranged oxalate crystals with associated tubular damage and interstitial inflammation, consistent with crystalline nephropathy. It is important to obtain a detailed history regarding over-the-counter supplement use in these patients, as early recognition allows for timely management, and such information may not be readily volunteered by patients. Prompt intervention and early management may prevent irreversible kidney damage, as evidenced by the partial recovery of renal function observed in our patient.

## Introduction

Vitamin D supplementation has intermittently been a topic of interest in the mainstream media over recent years, particularly during the COVID-19 pandemic, when it was widely suggested that maintaining adequate vitamin D levels was crucial for a strong immune system. Vitamin D is widely available in both pharmacies and regular supermarkets. With the rising availability and popularity of vitamin D supplements, clinicians are likely to encounter the effects of its excessive intake more frequently. While vitamin D toxicity may not initially be considered in the differential diagnosis of acute kidney injury, it is a recognised cause, particularly when accompanied by hypercalcaemia [1].

The current guidelines recommend a daily intake of 600-800 IU of vitamin D for adults to maintain adequate levels [2]. Brief periods of full-body exposure to midday summer sunlight are thought to produce sufficient amounts of vitamin D through skin synthesis, potentially as much as 10,000 IU per day [3]. However, while such endogenous production is subject to physiological regulation to prevent toxicity, excessive supplement intake, especially due to inappropriate prescribing or formulation errors, has been linked to toxic effects [4]. Clinicians should therefore be aware of how this toxicity presents, carefully review over-the-counter supplement use, and respond promptly in managing vitamin D-related renal injury.

## Case presentation

We present the case of a 71-year-old male who was referred to our tertiary renal centre from a district general hospital. He had originally presented with acute confusion, abnormal behaviour and generalised weakness, following a three-month history of declining mobility. On presentation, he appeared hypovolaemic, and blood tests revealed a significant acute kidney injury with a creatinine of 842 µmol/L, and a metabolic acidosis, with a pH of 7.18 and a bicarbonate of 17.8 mmol/L. He also had electrolyte abnormalities, including a high potassium of 5.8 mmol/L, a raised adjusted calcium of 3.56 mmol/L, and an elevated phosphate level at 2.65 mmol/L. His parathyroid hormone was suppressed at 4.0 pmol/L, and he had a normal alkaline phosphatase level. A myeloma screen was negative, with no paraprotein detected. Both kappa and lambda free light chains were elevated at 89.96 mg/L and 72.22 respectively, with a ratio of 1.25. He was also found to be anaemic with a haemoglobin of 70 g/L. Relevant laboratory values are presented in Table 1. CT of the abdomen revealed an 8-mm non‑obstructing calculus in the lower pole of the left kidney and a 3-mm calculus in the upper pole; flecks of calcification were seen scattered throughout the right kidney. He required haemodialysis for uraemia and persistent hyperkalaemia.

**Table 1:** Laboratory values at the time of patient's presentation Reference ranges are based on local laboratory standards eGFR: estimated glomerular filtration rate

Test	Observed value at presentation	Reference range
Urea (mmol/L or mg/dL)	36.6 mmol/L or 219.8 mg/dL	2.5-7.8 mmol/L or 15.0-46.8 mg/dL
Creatinine (µmol/L or mg/dL)	842 µmol/L or 9.5 mg/dL	65-104 µmol/L or 0.7-1.1 mg/dL
Potassium (mmol/L)	5.8	3.5-5.3
Sodium (mmol/L)	138	133-146
eGFR (ml/min/1.73m^2^)	5	>60
Bicarbonate (mmol/L)	17.8	22-29
pH	7.18	7.35-7.45
Adjusted calcium (mmol/L)	3.56	2.20-2.60
Phosphate (mmol/L)	2.65	0.80-1.50
Parathyroid hormone (pmol/L)	4	1.6-6.9
Alkaline phosphatase (U/L)	84	30-130
Haemoglobin (g/L or g/dL)	70 g/L or 7.0 g/dL	130-180 g/L or 13.0-18.0 g/dL
Kappa free light chains (mg/L)	89.96	6.7-22.4
Lambda free light chains (mg/L)	72.22	8.3-27.0
Kappa:lambda ratio	1.25	0.31-1.56
25-Hydroxyvitamin D (nmol/L or ng/mL)	521 nmol/L or 208.4 ng/mL	50-150 nmol/L or 20-60 ng/mL
Plasma oxalate (µmol/L)	8	<10
Urine oxalate excretion in 24 hours (µmol /24h)	401	80-490

The treating team initially ascribed his confusion and acute kidney injury to dehydration and the accumulation of his prescribed medications, gabapentin and gliclazide. During the admission, the patient’s daughter disclosed that he had been taking 20,000 IU of vitamin D each day for over three years, since the beginning of the COVID-19 pandemic. A sample of 25-hydroxyvitamin D was subsequently sent, and was elevated at 521 nmol/L, with a low parathyroid hormone, as seen in Table 1. Plasma oxalate concentrations were modestly elevated at 8 µmol/L, and urinary oxalate excretion was high-normal at 401 µmol/24 hrs. Despite haemodialysis and intravenous rehydration, the patient’s serum calcium remained elevated, and a single dose of intravenous pamidronate was administered. As the patient’s renal function had not recovered and he remained dialysis-dependent, a renal biopsy was performed.

**Figure 1:**
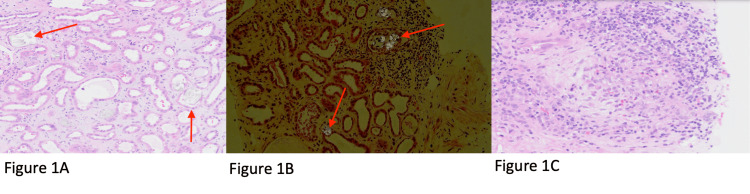
Renal biopsy showing crystalline nephropathy due to vitamin D intoxication Hematoxylin and eosin (H&E) stain at ×20 (1A, 1B, 1C). Birefringent crystals under polarised light (1B) demonstrate radially arranged birefringent oxalate crystals within renal tubules, tubular epithelium, and interstitium. There is associated interstitial oedema, tubular epithelial injury, and interstitial inflammation (1C)

Light microscopy of the sample demonstrated diffuse acute tubular injury with intratubular clear refractile crystals (Figure 1A). The crystals have a bright birefringence when viewed under polarised light (Figure 1B). The interstitium showed oedema and acute inflammatory infiltrate with prominent eosinophils and an ill-defined granuloma (Figure 1C).

The patient was commenced on prednisolone to suppress activation of vitamin D and to treat the interstitial nephritis, and counselled on the importance of good oral fluid intake. His renal function showed evidence of improvement, and dialysis was discontinued. Over subsequent months, his estimated glomerular filtration rate stabilised at ≈24 ml/min per 1.73 m^2^. His serum calcium also normalised, and his steroid dose was tapered down.

## Discussion

Vitamin D toxicity is a recognised but uncommon cause of hypercalcaemia and acute kidney injury [1], and supplementation is now increasingly driven by patient preference rather than medical advice. In our patient, the ingestion of 20,000 IU of vitamin D daily led to hypercalcaemia with subsequent parathyroid hormone suppression. Biochemical confirmation of this toxicity was demonstrated by a serum vitamin D level of 521 nmol/L. Markedly elevated serum 25-hydroxyvitamin D concentrations above 150 ng/ml (≈375 nmol/L), together with severe hypercalcaemia, hypercalcuria, and suppressed or undetectable parathyroid hormone levels, are hallmarks of vitamin D toxicity [5]. In our patient, it is likely that hypercalcaemia and hypercalciuria led to urinary supersaturation with calcium salts. This in turn led to deposition of calcium oxalate and phosphate crystals within the renal tubules and interstitium, subsequently causing tubular obstruction, interstitial injury, and a significant acute kidney injury [6,7].

Primary and secondary hyperoxaluria were excluded from our differentials due to the modest plasma oxalate level of 8 µmol/L and urinary oxalate excretion within the reference range. In primary hyperoxaluria, urinary oxalate excretion is generally >1.0 mmol/1.732 per 24 hours (≈1000 µmol/24 hr), with plasma oxalate often rising substantially as renal impairment worsens. In secondary hyperoxaluria, typically more modest increases are seen secondary to diet or intestinal absorption [8,9]. The oxalate crystal deposition in this case is therefore most consistent with calcium oxalate precipitation, secondary to vitamin D intoxication and hypercalcaemia. The histopathology in this case showed radially arranged oxalate crystals damaging the tubular epithelium with an associated interstitial inflammation (Figure 1), findings which are consistent with crystalline nephropathy. Similar patterns of renal injury have been observed in the context of vitamin D intoxication, as described by Herlitz et al. and Kumbar and Yee [6,7].

Management of vitamin D intoxication requires immediate discontinuation of the vitamin supplementation, alongside intravenous rehydration, dietary calcium restriction, and the use of loop diuretics. In cases of severe or persistent hypercalcaemia, bisphosphonate therapy or corticosteroids may be considered, as described by Marcinowska-Suchowierska et al. [5]. Renal replacement therapy may be necessary in cases with refractory hypercalcaemia or when acute kidney injury is severe [10]. Awareness of patients’ use of over-the-counter supplements is crucial, as they may not volunteer this information unless specifically asked. This is increasingly important due to more attention being drawn to self-supplementation of vitamin D by mainstream media. Early recognition of vitamin D intoxication allows for the prompt withdrawal of the offending agent and the commencement of supportive therapy, which could prevent irreversible kidney damage. The partial recovery of kidney function following cessation of excessive vitamin D intake in our patient underscores the importance of early recognition and prompt intervention in such cases.

## Conclusions

This report highlights the importance of careful history taking, including the use of over-the-counter and commercially available supplements, as excessive vitamin D intake can lead to hypercalcaemia, suppressed parathyroid hormone, and renal impairment. Hypercalcaemia leads to hypercalciuria and urinary supersaturation of calcium salts - subsequent deposition of calcium oxalate crystals in the tubular lamina causes tubulointerstitial nephritis and nephrocalcinosis. Treatment involves discontinuing exogenous vitamin D and ensuring adequate hydration, though in severe cases, bisphosphonates, corticosteroids, or even dialysis may be required. It is important to note that renal recovery may be partial and prolonged, as observed in our patient.
